# The Impact of Negative Symptoms and Neurocognition on Functioning in MDD and Schizophrenia

**DOI:** 10.3389/fpsyt.2021.648108

**Published:** 2021-07-26

**Authors:** Yue Feng Quek, Zixu Yang, Justin Dauwels, Jimmy Lee

**Affiliations:** ^1^Research Division, Institute of Mental Health, Singapore, Singapore; ^2^School of Electrical and Electronic Engineering, Nanyang Technological University, Singapore, Singapore; ^3^North Region & Department of Psychosis, Institute of Mental Health, Singapore, Singapore; ^4^Lee Kong Chian School of Medicine, Nanyang Technological University, Singapore, Singapore

**Keywords:** major depressive disorder, schizophrenia, negative symptoms, neurocognition, functioning

## Abstract

**Introduction:** Negative symptoms, neurocognitive deficits and functional impairment are prevalent in individuals with major depressive disorder (MDD) and schizophrenia (SCZ). However, unlike neurocognitive deficits, little is known about the role of negative symptoms toward functioning in individuals with MDD. On the other hand, both factors are well-studied in individuals with SCZ. Thus, this study aimed to examine the contributions of negative symptoms and neurocognitive impairments in functioning in individuals with MDD, compared to individuals with SCZ.

**Methods:** Participants included 50 individuals with MDD, 49 individuals with SCZ and 49 healthy controls. The following measures were administered—Negative Symptom Assessment (NSA-16), Brief Assessment of Cognition in Schizophrenia (BACS), Patient Health Questionnaire (PHQ-9), and MIRECC-Global Assessment of Functioning (MIRECC-GAF) to evaluate negative symptoms, neurocognition, depressive symptoms, and functioning respectively.

**Results:** Both MDD and SCZ groups had significantly more severe negative symptoms, depressive symptoms, and poorer functioning than healthy controls. Individuals with SCZ performed significantly poorer on the BACS than the other two groups. Both negative symptoms and neurocognition were significantly correlated with social and occupational functioning in SCZ. Motivation subdomain of the negative symptoms was significantly correlated with occupational functioning, while depressive symptoms correlated with functioning in MDD.

**Conclusion:** Both negative symptoms and neurocognitive deficits appear to play differential roles on individual domains of functioning between MDD and SCZ. Future longitudinal studies with larger sample sizes should be done for a better understanding about the associations between the factors and functioning.

## Introduction

According to the World Health Organisation (WHO) (1), MDD is a leading cause for non-fatal health loss and has prevailed as one for nearly three decades. With varying degrees of severity and scope, most individuals with major depressive disorder (MDD) face some forms of impairment in their daily functioning, such as work or interpersonal relationships (2). Studies have reported that only 20% of individuals with MDD manage to attain complete functional recovery (3, 4). The persistence of functional disruption, even in symptomatic remission, highlights the insufficiency of symptom reduction as an end goal for individuals with MDD. A return to premorbid level of functioning remains a key treatment goal (5). As such, it is important to understand factors that influence functioning in order to provide the right treatment to people with MDD and aid them in their functional recovery.

In the literature, two factors—neurocognitive deficits and negative symptoms often stand out in relation to their strong association with functioning. Specifically in MDD, neurocognitive deficits are prevalent in the individuals and have been reported to be one of the core features of the condition (6, 7). These deficits often involve multiple subdomains of cognitive functions and are heterogeneous between individuals (8, 9). Strong and consistent associations have been demonstrated linking neurocognitive deficits to functional impairment in individuals with MDD (7, 10). Another study (11) also found that baseline cognitive deficits was significantly associated with functional disability measured 6 months later. In addition, the authors found that improvements in neurocognition was associated with greater functional recovery over the course of follow up. These results support the role of neurocognitive deficits in functional recovery for individuals with MDD.

Negative symptoms, i.e., asociality, amotivation, affect blunting and alogia, commonly described in schizophrenia are less studied in MDD population (12). While studies (13–15) have provided substantial evidence about the presence of negative symptoms in this population, the relationship between negative symptoms and functioning in MDD is however sparely addressed. Two studies (16, 17) have found motivation-related deficits to be significantly related to functioning. Another study (18) also found that loss of interest was associated with work and family life impairment. However, the authors had classified loss of interest as part of *residual depressive symptoms* alongside sleeping and concentration difficulties, rather than as a construct of negative symptoms. While depressive symptoms have a considerable amount of overlap with negative symptoms (12, 19), both concepts are often suggested to be separable and independent concepts (12, 14). On the other hand, there were contrary old findings (20) where no significant associations between negative symptoms and role functioning were observed. In that study, the authors had studied negative symptoms in three specific domains—flat affect, alogia, and motor retardation. This incongruence might suggest that specific negative symptoms domains have differential effects on functioning in MDD. Therefore, the role of negative symptoms in functional impairment in individuals with MDD remains unclear and require further examinations.

On the other hand, both neurocognitive deficits and negative symptoms have been widely studied and can be considered the most significant correlates of impaired functioning in individuals with schizophrenia (SCZ) (21–24). Few studies have compared the predictive abilities of both factors; some studies reported neurocognitive deficits to be superior (25, 26) while some found otherwise (27). However, they support the contribution of both factors toward functional impairment in SCZ.

Therefore, the aim of the current study is to better understand negative symptoms and neurocognitive deficits in MDD in their individual contribution toward functioning, alongside individuals with SCZ and healthy individuals.

## Methods

### Participants

Fifty individuals with MDD and 49 individuals with SCZ were recruited from the Institute of Mental Health, Singapore. Forty-nine healthy controls (HC) were recruited from the community. The data collection was completed between September 2017 and April 2019.

The inclusion criteria for the patient groups were as follows: diagnosis of SCZ or MDD, aged 21–65 years, English-speaking and has capacity to provide informed consent. The inclusion criteria for the HC group includes aged 21–65, English-speaking, has capacity to provide informed consent and no history of any mental disorder. Individuals with intellectual disability, neurological disorders, and history of cerebrovascular accidents or traumatic brain injuries were excluded.

The diagnoses of SCZ and MDD were ascertained on the Structured Clinical Interview for DSM-IV (SCID-I/P) (28); HCs were screened using the non-patient version (SCID-I/NP) (29).

### Procedure

All the assessments were conducted by trained research psychologists in a single visit. In order to reduce bias in rating negative symptoms, the assessments for negative symptoms were conducted by a research psychologist who was not involved in the SCID interview and blinded to the participant's condition. This study was reviewed and approved by the National Healthcare Group Domain Specific Review Board (DSRB), Singapore. Written informed consent was obtained from all participants.

### Measures

#### Negative Symptoms

The 16-item Negative Symptom Assessment (NSA-16) (30, 31) was used to assess the negative symptoms in all participants. The NSA-16 is a semi-structured interview which assesses negative symptomology in the past 7 days. There are 16 items in the scale, each scored on a 6-point scale. A 7-point global negative symptom rating is also scored, assessed based on the interviewer's overall gestalt of the severity of the individual's negative symptoms. Higher scores in the scale reflect higher severity of negative symptoms. Five symptom factors—Emotion/affect, communication, social involvement, motivation, and retardation were derived from factor analysis (31) and used in the analysis. The NSA-16 has a high Cronbach's alpha of 0.92 (31).

#### Neurocognition

The Brief Assessment of Cognition in Schizophrenia (BACS) (32) was used to assess the cognitive abilities of the participants. The BACS consists of six tasks that evaluate the cognitive domains that are persistently impaired and strongly associated with functional outcomes in individuals with SCZ—Verbal memory, working memory, motor speed, attention, executive functioning, and verbal fluency. The BACS has been shown to have high test-retest reliability in individuals with SCZ and controls (32). *Z*-scores of each subtest were computed based on the results from the original developers and a composite score was then obtained by averaging the z-scores of the six subtests (32). BACS has also been used in other studies to assess cognitive abilities in MDD (33–35) and has shown its ability to differentiate cognitive abilities between individuals with MDD from HC (8).

#### Depressive Symptoms

The Patient Health Questionnaire-9 (PHQ-9) (36, 37) was used to assess severity of depressive symptoms in the participants. PHQ-9 is a patient-reported questionnaire which used the criteria from the DSM-IV symptoms for MDE. The scale consists of 9 items that are each scored on a frequency scale from 0 to 3, 0 being not at all and 3 being nearly every day. It also consists of a tenth item that rates the patient's difficulty in functioning, which is not used for scoring. PHQ-9 has been found to have good psychometric properties (36).

#### Functioning

The MIRECC-Global Assessment of Functioning (MIRECC-GAF) (38) was used to assess the real-world functioning of the participants. This clinician-rater scale measures the participants' occupational functioning, social functioning and symptom severity on three individual subscales. The range of the scores on each subscale is from 0 to 100, with higher scores reflecting better functioning or lower symptom severity. The developers have reported that MIRECC-GAF has high reliability (ICCs >0.98), predictive abilities, and superior concurrent validity than the conventional GAF scores (38).

### Statistical Analysis

Normality assumptions and homogeneity of variance were tested using Shapiro-Wilk and Levene's tests respectively. Transformation was performed on those variables that were not normally distributed. The NSA-16 subdomains—social involvement in SCZ and motivation in MDD groups were normally distributed after applying square root transformation. All three MIRECC-GAF domains in MDD and SCZ groups were normally distributed after applying Blom's transformation. However, for the three MIRECC-GAF domains in HC, departure from normality was severe and none of the transformation method could yield a normal distribution. Parametric tests were used for normally-distributed variables while non-parametric tests were used when non-normally distributed variables were involved. For comparisons between the three diagnostic groups, the Chi-squared tests were used for categorical variables; the Univariate Analysis of Variance (ANOVA) was used for continuous variables if normality assumption was met, while the Kruskal-Wallis H test was used if the normality assumption was violated. Correlation analysis was used to measure the association between NSA-16, BACS, PHQ-9, and MIRECC-GAF. The NSA-16 total scores were used in this study. The analyses with NSA-16 global scores are available in the Supplementary Table 1. The data was analyzed using IBM SPSS version 23 (39).

## Results

### Demographics and Clinical Characteristics

The demographics and clinical characteristics of the participants are shown in Table 1. There were no statistical differences between the groups in terms of gender, employment status, age and total years of education. Significantly more HCs (57.1%) are married as compared to the MDD (24%) and SCZ (14.3%) groups. The SCZ group (*M* = 14.30, *SD* = 10.22) has significantly longer duration of illness than the MDD group (*M* = 4.80, *SD* = 4.94).

**Table 1:** Demographic and clinical characteristics.

**Variable**	**MDD**	**SCZ**	**HC**	**Statistic value**	***p***
	**(*****n*** **= 50)**	**(*****n*** **= 49)**	**(*****n*** **= 49)**		
Male	26 (52.0)	25 (51.0)	26 (53.1)	$\chi_{\left(2\right)}^{2}$ = 0.04	0.980
Married	12 (24.0)	7 (14.3)	28 (57.1)	$\chi_{\left(2\right)}^{2}$ = 22.04	<0.001
Employed	33 (66.0)	30 (61.2)	34 (69.4)	$\chi_{\left(2\right)}^{2}$ = 0.73	0.694
Ethnicity				$\chi_{\left(2\right)}^{2}$ = 15.56	0.016
Chinese	36 (72.0)	41 (83.7)	32 (65.3)		
Malay	5 (10.0)	2 (4.1)	13 (26.5)		
Indian	6 (12.0)	6 (12.2)	3 (6.1)		
Other	3 (6.0)	0 (0.0)	1 (2.0)		
Age in years	36.66 ± 13.01	40.53 ± 10.42	39.98 ± 12.41	*H*_(2)_ = 4.16	0.125
Total years of education	14.20 ± 2.60	13.50 ± 2.86	13.54 ± 2.90	*F*_(2,145)_ = 0.97	0.382
Duration of illness (in years)	4.80 ± 4.94	14.30 ± 10.22	NA	*U* = 459.50	<0.001
MIRECC-GAF					
Symptomatic	50.02 ± 17.43	55.16 ± 16.20	90.90 ± 7.62	*H*_(2)_ = 64.94	<0.001
Social	63.56 ± 10.41	62.94 ± 11.99	81.94 ± 8.17	*H*_(2)_ = 65.76	<0.001
Occupational	55.72 ± 23.25	53.16 ± 23.14	87.39 ± 7.47	*H*_(2)_ = 94.48	<0.001
BACS Z-score					
Verbal memory	−0.01 ± 1.11	−0.76 ± 1.25	−0.11 ± 1.02	*F*_(2,145)_ = 6.39	0.002
Digit sequencing	−0.03 ± 1.11	−0.61 ± 1.23	−0.10 ± 1.10	*H*_(2)_ = 6.83	0.033
Token motor task	−0.56 ± 1.02	−1.41 ± 1.19	−0.30 ± 1.00	*H*_(2)_ = 21.92	<0.001
Semantic	0.36 ± 1.19	−0.84 ± 0.87	0.21 ± 1.19	*H*_(2)_ = 29.47	<0.001
fluency (Total)					
Symbol coding	−0.40 ± 1.23	−1.55 ± 1.23	−0.29 ± 1.13	*H*_(2)_ = 21.91	<0.001
Tower of London	0.17 ± 0.77	−0.56 ± 1.62	−0.03 ± 1.05	*H*_(2)_ = 5.80	0.055
Composite	0.13 ± 1.19	−1.60 ± 1.59	−0.17 ± 1.21	*F*_(2,145)_ = 19.30	<0.001
NSA-16					
Communication	7.02 ± 2.33	8.00 ± 3.27	6.14 ± 2.39	*H*_(2)_ = 11.76	0.003
Emotion/Affect	8.46 ± 2.29	8.96 ± 2.39	7.86 ± 1.98	*H*_(2)_ = 6.03	0.049
Social involvement	8.30 ± 2.64	8.82 ± 2.72	7.04 ± 2.27	*H*_(2)_ = 11.26	0.004
Motivation	12.14 ± 2.70	11.61 ± 2.63	7.78 ± 2.47	*H*_(2)_ = 54.48	<0.001
Motor retardation	4.46 ± 1.89	4.59 ± 2.06	3.08 ± 1.24	*H*_(2)_ = 19.00	<0.001
Total	40.38 ± 7.79	41.98 ± 9.87	31.90 ± 7.07	*H*_(2)_ = 37.52	<0.001
Global impression	3.50 (4)	3 (3)	2 (3)	*H*_(2)_ = 62.47	<0.001
score^*^					
PHQ-9	14.02 (7.00)	6.84 (5.92)	2.55 (2.99)	*H*_(2)_ = 63.23	<0.001

*Data are expressed as mean ± SD or n (%)*.

* *Global impression score is reported as median (range)*.

*MIRECC-GAF, MIRECC-Global Assessment of Functioning; BACS, Brief Assessment of Cognition in Schizophrenia; NSA-16, Negative Symptom Assessment. PHQ-9, Patient Health Questionnaire*.

Within the MDD group, males (*M* = 62.77, *SD* = 24.16) were found to have significantly higher MIRECC-GAF occupational functioning scores than females (*M* = 48.08, *SD* = 20.00), *T*_(47)_ = 2.17, *p* = 0.035. None of the other socio-demographic factors were found to be significantly associated with the three MIRECC-GAF domains.

With regards to the clinical characteristics between groups, the SCZ group performed significantly poorer on BACS composite score than the other two groups. Both the SCZ and MDD groups had significantly poorer MIRECC-GAF ratings, higher NSA-16 total, subdomains, global scores, and PHQ-9 total than the HCs.

### Associations Between Cognition, Negative Symptoms, Depressive Symptoms, and Functioning Across Groups

Univariate analyses were performed to test whether socio-demographic factors including age, gender, duration of illness and total years of education were associated with functioning in each diagnostic group. Only gender was significantly associated with occupational functioning in MDD.

The results of correlational analyses between cognition, negative symptoms, depressive symptoms, and functioning across groups are presented in Tables 2, 3 and Supplementary Table 3. For individuals with MDD, NSA-16 total was significantly associated with the MIRECC-GAF symptomatic (*r* = −0.39, *p* = 0.005). Within the subdomains of NSA-16, motivation was significantly correlated with the MIRECC-GAF occupational functioning (*r* = −0.48, *p* < 0.001) and symptomatic functioning (*r* = −0.42, *p* = 0.003). Another subdomain, motor retardation was significantly associated with the MIRECC-GAF symptomatic subscale (*r* = −0.38, *p* = 0.007). No significant association was observed between BACS composite score and MIRECC-GAF subscales. The 6 individual BACS domains were also not significantly associated with the MIRECC-GAF subscales (details were presented in Supplementary Table 2). PHQ-9 score was found to be significantly correlated with MIRECC-GAF social (*r* = −0.54, *p* < 0.001) and symptomatic (*r* = −0.50, *p* < 0.001) domains. In view of the significant relationship of gender on occupational functioning in MDD, a multiple regression for occupational functioning was performed using gender and NSA-16 motivation as predictors (*F* = 11.35, *p* < 0.001); this revealed a significant effect of NSA-16 motivation (β = −0.18, *p* < 0.001) on occupational functioning in MDD.

**Table 2:** Association between MIRECC-GAF, BACS composite, NSA-16 total and PHQ-9 total across groups.

**Measure**	**MDD**			**SCZ**			**HC**		
	**Occ**	**Soc**	**Symp**	**Occ**	**Soc**	**Symp**	**Occ**	**Soc**	**Symp**
BACS	0.22^a^	0.12^a^	0.001^a^	0.41^**^^a^	0.25^a^	0.39^**^^a^	0.14	0.17	0.08
NSA-16	−0.19^a^	−0.19^a^	−0.39^**^^a^	−0.32^*^^a^	−0.36^*^^a^	−0.20^a^	0.08	−0.17	−0.07
PHQ-9	−0.07^a^	−0.54^**^^a^	−0.50^**^^a^	−0.18	−0.11	−0.32^*^	−0.10	−0.21	−0.15

* *p < 0.05,*

** *p < 0.01.*

a *Pearson's correlation*.

*BACS, Brief Assessment of Cognition in Schizophrenia; NSA-16, Negative Symptom Assessment; PHQ-9, Patient Health Questionnaire; Occ, MIRECC-GAF Occupational functioning; Soc, MIRECC-GAF Social functioning; Symp, MIRECC-GAF Symptomatic functioning*.

**Table 3:** Correlations between MIRECC-GAF and NSA-16 domains between groups.

**NSA-16 domains**	**MDD**			**SCZ**			**HC**		
	**Occ**	**Soc**	**Symp**	**Occ**	**Soc**	**Symp**	**Occ**	**Soc**	**Symp**
Communication	−0.001^a^	−0.19^a^	−0.24^a^	−0.26^a^	−0.42^**^^a^	−0.33^**^^a^	−0.04	−0.05	−0.11
Emotion/Affect	0.002^a^	−0.003^a^	−0.21^a^	−0.18^a^	−0.17^a^	−0.11^a^	0.38^**^	−0.03	0.12
Social involvement	−0.07^a^	−0.12^a^	−0.08^a^	−0.09^a^	−0.32^*^^a^	−0.03^a^	0.01	−0.26	0.06
Motivation	−0.48^**^^a^	−0.23^a^	−0.42^**^^a^	−0.49^**^^a^	−0.28^a^	−0.18^a^	0.11	−0.29^*^	−0.03
Motor retardation	−0.05^a^	−0.11^a^	−0.38^**^^a^	−0.18^a^	−0.05^a^	−0.06^a^	0.06	0.09	−0.14

* *p < 0.05,*

** *p < 0.01.*

a *Pearson's correlation*.

*NSA-16, Negative Symptom Assessment; Occ, MIRECC-GAF Occupational functioning; Soc, MIRECC-GAF Social functioning; Symp, MIRECC-GAF Symptomatic functioning*.

For individuals with SCZ, both the NSA-16 total and BACS composite score were significantly correlated with most MIRECC-GAF subscale scores. Within the NSA-16 subdomains, motivation was significantly correlated with the MIRECC-GAF occupational functioning (*r* = −0.49 *p* < 0.001), while social involvement was associated with MIRECC-GAF social functioning (*r* = −0.32, *p* = 0.24). Communication was significantly correlated with two MIRECC-GAF subscales—Social (*r* = −0.42, *p* = 0.002) and Symptomatic (*r* = −0.33, *p* = 0.002). PHQ-9 score was significantly correlated with only MIRECC-GAF symptomatic domain (Spearman's rho = −0.32, *p* = 0.026).

In HC, neither the BACS nor NSA-16 total scores were significantly correlated with the MIRECC-GAF score. However, two of the NSA-16 subdomains—emotion/affect and motivation were significantly associated with the MIRECC-GAF occupational (Spearman's rho = −0.38, *p* = 0.007) and social (Spearman's rho = −0.29, *p* = 0.045) subscales respectively. PHQ-9 score was not significantly correlated with MIRECC-GAF.

## Discussion

This study examined the association of negative symptoms, neurocognitive deficits, and depressive symptoms with functioning in individuals with MDD, individuals with SCZ and HC. The results revealed differences across the three groups in terms of severity of neurocognitive deficits, negative symptoms, depressive symptoms and functioning. The MDD and SCZ groups had more severe depressive ratings than the HC, while the SCZ group had greater impairments in neurocognition compared to the two other groups, consistent with existing studies (28, 29, 40). Both the MDD and SCZ groups had significantly greater negative symptom severity and poorer functioning than HC, which is consistent with the current literature (2, 13, 40, 41). The relationships between the NSA-16 subdomains differed across the three groups. In MDD, the NSA-16 motivation subdomain was significantly associated with occupational functioning. On the other hand, depressive symptoms were associated with social functioning, which is consistent with existing literature (42, 43). Both motivation subdomain and depressive symptoms were also associated with symptomatic functioning. On the other hand, in SCZ, neurocognition was associated with occupational and symptomatic functioning while overall negative symptoms and some of the subdomains were associated with all three domains of functioning. As for the HC, negative symptom subdomains, specifically emotion/affect and motivation, were significantly associated with occupational and social functioning. Neurocognition was not significantly associated with functioning in MDD and HC.

The results have implications on the presence and nature of negative symptoms in individuals with MDD. Negative symptoms are present in MDD, almost to the same degree of severity as individuals with SCZ, as seen in the lack of significant difference in the negative symptom subdomains and total severity rating between both groups. This is consistent with existing studies (44, 45), which have shown comparable severity between both groups. This further highlights the importance of assessing and treating negative symptoms in MDD in clinical treatment.

This study found that the overall negative symptoms severity was not significantly associated with functioning in MDD; only the motivation subdomain was associated with occupational functioning in MDD. Though the impairments in negative symptoms are present across multiple subdomains, the subdomains are however, not equal in terms of their impact on functioning. This further suggests that treatments that target functional recovery in MDD should focus on motivation in order to ensure optimal results. Furthermore, the differential associations between SCZ and MDD suggest that specific subdomains of negative symptoms are related to different domains of functioning in the two diagnostic groups. Only motivation appears to be consistently associated with occupational functioning in both groups. This finding has also been seen in other studies that examined both groups individually (16, 46). A possible explanation for the link between motivation and occupational functioning could be the individuals having aberrant cost-benefit calculations as a result of neural abnormalities in reward-processing circuit (47), which leads them to choose passive tasks that mostly require lesser effort e.g., laying on bed instead of performing active tasks like going out to work or doing housework when at home. There had been some focus in explicitly targeting rewards in psychosocial treatments in individuals with MDD, with a goal of providing the individuals with more exposure to rewarding elements and personal experience of reward (48). A review (49) has found that such explicit behavioral exposure has benefits on clinical outcomes in the individuals. A more recent study has also found that reward exposure therapy through behavioral activation helped reduce depressive symptoms in a group of older adults with MDD (50). With this positive finding, it might be worth studying if such benefits could also be broadened onto the individuals' functioning abilities.

Unexpectedly, this study did not find a significant association between neurocognitive deficits and functioning in MDD, which is inconsistent with the current literature (6, 10, 11). A potential explanation would be relevant to the relatively younger ages of the MDD participants in the study, where almost half the group (46%) is aged 30 years old and below. It is also evident in the significantly shorter duration of illness in the MDD group. Neurocognitive abilities tend to deteriorate with age (51), and studies have found that neurocognitive impairments are typically less pronounced in younger adults with MDD (52). As such, this suggests that the neurocognitive impairments in this current sample of MDD might have been minimal, as backed by the lack of significant difference in BACS performance between them and the HC. Hence, the seemingly absent neurocognitive deficits faced by the younger MDD group might have been the reason for the minimal association with functioning seen in this study. On the other hand, neurocognitive impairments were significantly associated with occupational functioning in the SCZ group. This difference between both groups can thus be attributed to the younger MDD group, and the relationship between neurocognitive impairments and functioning being age-dependent in MDD, unlike that in SCZ (53).

This study provided more information on the effects of negative symptoms on functioning in MDD, an area that has not been studied widely (54, 55). One strength of this study is that the raters who performed NSA-16 were blinded to the diagnosis of the participants. This would reduce rating bias related to rater's preconceived notions of negative symptoms in MDD or SCZ. However, there are also limitations worthy of mention. First, this is a cross-sectional study, and findings reported are only associational in nature. As such, we were not able to examine the trajectories of negative symptoms and cognitive impairments in MDD and its relationship to functioning. Second, NSA-16 is a measure that is validated in SCZ and its validity in MDD might not have been previously reported. Also, NSA-16 does not directly address anhedonia, one of the negative symptom subdomains (56). A past study (57) has found that anhedonia is a strong predictor of psychosocial functioning in depressed patients. That said, the objective of the study requires a common scale to be used across all groups so that comparisons can be made. Future studies might seek to address these issues related to the properties of the measures. Third, medication use might have effects on the variables of interest, e.g., benzodiazepine and antidepressants use were found to affect different cognitive domains (8). However, due to the small sample size in this study, the effects of medications were not studied. Lastly, the short duration of illness for the MDD group might affect the generalizability of the study results. The relationship between negative symptom and neurocognition with functioning may vary as a function of illness duration. Future studies may seek to examine the relationship between factors with a sample of a larger range of illness duration for better generalizability.

## Conclusion

In this study, we reported the presence of negative symptoms in individuals with MDD. Also, specific negative symptoms subdomains and neurocognitive deficits were found to play different roles in individual domains of functioning between MDD and SCZ. Having validation studies on transdiagnostic measures might be useful for interpreting results of future MDD studies. Additionally, future longitudinal studies with larger sample sizes would improve our understanding about the relationship between the factors and functioning in MDD and SCZ.

## Data Availability Statement

The original contributions presented in the study are included in the article/Supplementary Material, further inquiries can be directed to the corresponding authors.

## Ethics Statement

The studies involving human participants were reviewed and approved by NHG Domain Specific Review Board (DSRB). The patients/participants provided their written informed consent to participate in this study.

## Author Contributions

YQ has been involved in the data entry, data analysis, drafting and revision of the manuscript, and the final approval of the final submission. ZY has been involved in the conception and design of research study, writing the protocol, data collection, data entry, data analysis, revision of the manuscript, and final approval of the final submission. JD has been involved in the conception and design of research study, and final approval of the final submission. JL has been involved in the conception and design of research study, data collection, data analysis, revision of the manuscript and final approval of the final submission. All authors contributed to the article and approved the submitted version.

## Conflict of Interest

The authors declare that the research was conducted in the absence of any commercial or financial relationships that could be construed as a potential conflict of interest.

## Publisher's Note

All claims expressed in this article are solely those of the authors and do not necessarily represent those of their affiliated organizations, or those of the publisher, the editors and the reviewers. Any product that may be evaluated in this article, or claim that may be made by its manufacturer, is not guaranteed or endorsed by the publisher.
