# Supplementary material for: The Impact of Negative Symptoms and Neurocognition on Functioning in MDD and Schizophrenia
Source: Front Psychiatry. 2021 Jul 26;12:648108. doi: 10.3389/fpsyt.2021.648108 (PMC8350050; doi:10.3389/fpsyt.2021.648108)
Supplement: Supplementary file 1 [file Table_1.pdf]

Table 1. Association between MIRECC-GAF and NSA-16 global impression scores

MIRECC-GAF components	MDD	SCZ	HC
Occupational	-0.30*	-0.32*	0.02
Social	-0.29*	-0.44**	-0.18
Symptomatic	-0.46**	-0.29*	-0.02

Data was analysed using Spearman's rank correlation; *$p < 0.05$, **$p < 0.01$

Abbreviations: MIRECC-Global Assessment of Functioning, MIRECC-GAF
