# Supplementary material for: The Impact of Negative Symptoms and Neurocognition on Functioning in MDD and Schizophrenia
Source: Front Psychiatry. 2021 Jul 26;12:648108. doi: 10.3389/fpsyt.2021.648108 (PMC8350050; doi:10.3389/fpsyt.2021.648108)
Supplement: Supplementary file 2 [file Table_2.pdf]

Table 2. Association between MIRECC-GAF functioning components and BACS components

BACS components	MDD			SCZ			HC		
	Occ	Soc	Symp	Occ	Soc	Symp	Occ	Soc	Symp
Verbal memory	0.08 [a]	0.11 [a]	0.14 [a]	0.35* [a]	0.22 [a]	0.32* [a]	0.23	0.19	-0.06
Digit sequencing	0.18 [a]	0.13 [a]	0.07 [a]	0.28 [a]	0.09 [a]	0.42** [a]	0.13	0.10	0.15
Token motor task	0.19 [a]	-0.12 [a]	-0.21 [a]	0.25 [a]	0.03 [a]	0.21 [a]	0.10	0.23	0.19
Semantic fluency	0.07 [a]	0.14 [a]	-0.07 [a]	0.32* [a]	0.18 [a]	0.26 [a]	0.001	0.16	0.08
Symbol coding	0.23 [a]	0.07 [a]	-0.04 [a]	0.45** [a]	0.33* [a]	0.27 [a]	0.01	-0.06	0.01
Tower of London	0.09[a]	0.18 [a]	0.17 [a]	0.26 [a]	0.26 [a]	0.30* [a]	0.07	-0.11	-0.01

*$p < 0.05$, **$p < 0.01$

Note: [a] Pearson's correlation

Abbreviations: Brief Assessment of Cognition in Schizophrenia, BACS; MIRECC-GAF Occupational functioning, Occ; MIRECC-GAF Social functioning, Soc; MIRECC-GAF Symptomatic functioning, Symp
