# Supplementary material for: The Impact of Negative Symptoms and Neurocognition on Functioning in MDD and Schizophrenia
Source: Front Psychiatry. 2021 Jul 26;12:648108. doi: 10.3389/fpsyt.2021.648108 (PMC8350050; doi:10.3389/fpsyt.2021.648108)
Supplement: Supplementary file 3 [file Table_3.pdf]

Table 3. Association between PHQ-9 and NSA-16

NSA-16 domains	MDD	SCZ	HC
Communication	0.18 [a]	0.28 [a]	0.34*
Emotion/Affect	0.17 [a]	0.08 [a]	-0.11 [a]
Social involvement	0.02 [a]	0.19 [a]	-0.08
Motivation	0.36** [a]	0.16 [a]	0.09 [a]
Motor retardation	0.25 [a]	0.20 [a]	0.03 [a]
Total	0.29* [a]	0.25 [a]	0.15

*$p < 0.05$, **$p < 0.01$

Note: [a] Pearson's correlation

Abbreviations: Negative Symptom Assessment, NSA-16; Patient Health Questionnaire, PHQ-9
